# Selective trimerization of ethylene using chromium catalysts complexed with tridentate ligands supported on titanium modified silica

**DOI:** 10.1038/s41598-025-02844-9

**Published:** 2025-05-25

**Authors:** Younes Habibi, Mohamadreza Marefat, Sajjad Gharajedaghi, Masoumeh Mohamadhoseini, Zahra Mohamadnia, Ebrahim Ahmadi

**Affiliations:** 1 Department of Chemistry, Institute for Advanced Studies in Basic Science (IASBS), Gava Zang, Zanjan, 45137-66731 Iran; 2 Department of Chemistry, University of Zanjan, P.O. Box 45195-313, Zanjan, Iran

**Keywords:** Ethylene trimerization, 1-Hexene, Heterogeneous catalyst, SBA-15, Titanium dioxide, Chromium catalyst, Chemical engineering, Nanoscale materials

## Abstract

Alpha-olefins like 1-hexene play a crucial role in petrochemical industries. This study investigates the development of chromium-based catalysts featuring tridentate ligands for selective 1-hexene production via ethylene trimerization under optimized conditions: 80 °C, 25 bar ethylene, 40 mL anhydrous toluene, and MMAO (Al/Cr = 700/1) as the co-catalyst. Both homogeneous catalysts (Cr-SNS-D and Cr-SNS-B) and heterogeneous catalysts supported on SBA-15, including titanium-modified SBA-15 (Ti-SBA-15), were examined. The homogeneous catalysts exhibited remarkable selectivity (99.9%) and high catalytic activity, reaching 60,772 and 53,344 g 1-C₆ g Cr⁻¹ h⁻¹, respectively. Upon immobilization onto SBA-15 supports, a slight reduction in activity was observed; however, the introduction of titanium into the SBA-15 framework led to a 1.5-fold enhancement in catalytic performance (35,115 g 1-C₆ g Cr⁻¹ h⁻¹) and a significant reduction in polyethylene by-product formation. This study highlights the critical influence of support modification on catalytic behavior, demonstrating how tailored electronic interactions between the active sites and the support can optimize performance. The findings provide a valuable framework for designing more efficient chromium-based catalysts, bridging the gap between homogeneous and heterogeneous systems for improved 1-hexene production.

**Supplementary Information:**

The online version contains supplementary material available at 10.1038/s41598-025-02844-9.

## Introduction

Linear alpha-olefins (LAOs) are a prominent product of ethylene oligomerization. The conversion of ethylene into larger oligomers, a process known as olefin oligomerization, has garnered significant attention from scientists and industry professionals. With the increasing production of polyolefins, the demand for linear alpha-olefins as monomers has surged. These LAOs are integral in the manufacturing of polyethylene, synthetic lubricants, plasticizer alcohols, surfactants, and various other chemical products^1,2^. Ethylene oligomerization is typically conducted using intermediate metal-based catalysts such as zirconium (Zr)^3^, chromium (Cr)^4–6^, vanadium (V)^7^, and titanium (Ti)^8^. These catalysts produce a broad spectrum of linear alpha-olefins (LAOs) in a Schulz-Flory distribution with various chain lengths. However, this diversity of products often does not meet commercial demand. Among these catalytic systems, chromium-based catalysts stand out for their exceptional selectivity and activity in producing 1-hexene, a process that has already been scaled up for industrial applications^9,10^. One of the primary objectives of ethylene oligomerization is the formation of 1-hexene and 1-octene^11,12^. This goal has attracted significant interest from major companies worldwide, including Sasol, SABIC, Mitsui, Chevron Phillips Chemical Company, and Nova Chemicals^13,14^. More than 90% of patents and scientific papers related to the ethylene trimerization highlight the use of chromium-based catalysts^15,16^. Consequently, the chromium catalytic system is particularly well-suited for investigating the factors that influence the selectivity and activity of ethylene oligomerization and polymerization. While homogeneous catalysts are commonly employed in commercial ethylene oligomerization processes, green chemistry principles advocate for the use of heterogeneous catalysts as a more convenient and environmentally sustainable approach. Heterogeneous catalysts offer several advantages, including easier separation from reaction mixtures, reduced waste generation, and enhanced catalyst recyclability, aligning with the goals of green chemistry to minimize environmental impact and improve process efficiency^17,18^. These attributes make heterogeneous catalysts a promising alternative for more sustainable and efficient chemical processes^19,20^. While numerous heterogeneous catalysts have been synthesized and utilized in organic chemistry, their application in ethylene trimerization reactions has been limited^21,22^. Homogeneous catalysts for ethylene trimerization frequently result in the formation of undesirable polyethylene as a byproduct. The accumulation of polyethylene residue within the reactor can significantly disrupt mechanical processes, resulting in reactor fouling. To minimize reactor fouling, it is crucial to reduce the presence of unwanted polyethylene during ethylene oligomerization or trimerization. Using heterogeneous catalysts with high selectivity in the oligomerization of olefins provides a constructive approach to reduce reactor fouling. Heterogeneous catalysts can be prepared by physically or covalently immobilizing activated catalysts onto or into a support surface^23,24^. The stabilization of transition metals onto supports has a significant impact on traditional solution-phase chemistry. This stabilization can be achieved on various substrates, including mesoporous silica materials^25^, metal-organic frameworks^26^, zeolites^27^, and polymeric supports^28^. These supports enhance the performance and durability of the catalysts, contributing to more efficient and sustainable chemical processes.

In 2024, our research team modified mesoporous silica (MS) with ionic liquids (ILs) containing BF_4_^−^ and Br^−^ counter-anions to create IL-BF_4_@MS and IL-Br@MS, respectively. We then synthesized twelve catalysts by immobilizing half-sandwich catalysts with various bridges onto the surfaces of MS, IL-Br@MS, and IL-BF_4_@MS. Utilizing these immobilized catalysts simplifies the purification process, enabling the isolation of pure products. This methodology not only improve catalytic activity but also increases the overall efficiency of the trimerization process by reducing the complexity of product separation^24^. In 2017, Fallahi et al. introduced an innovative catalyst by anchoring bis(2-alkylsulfanylethyl)amine-CrCl_3_ onto silica modified with an ionic liquid. The incorporation of an imidazolium-based ionic liquid onto the mesoporous silica not only served as an effective strategy to shield surface hydroxyl groups but also provided a platform to support BF_4_^−^, a known promoter for chromium-based catalysts. This Cr-SNS-IL(BF_4_)@MS catalyst demonstrated remarkable performance, achieving an activity of 51,079 and a selectivity exceeding 99% ^29^. In 2020, we also examined the effects of ionic liquid counter anions on the selectivity and efficiency of Cr-SNS-R-based ethylene trimerization catalysts. This research aimed to elucidate how various anions influence catalytic performance and product distribution, offering insights into optimizing catalyst formulations for improved efficiency and selectivity in ethylene trimerization process^30^. One approach of preparing heterogeneous systems involves stabilizing transition metal catalysts on mineral oxides. In 2019, Goetjen et al.^31^ introduced NU-1000, a heat-resistant metal-organic framework (MOF), as a novel heterogeneous catalytic system for ethylene oligomerization. This innovative catalyst demonstrated remarkable thermal stability and efficacy in promoting the oligomerization process. Also, In 2024, Jiang et al.^32^ utilized density functional theory (DFT) calculations to analyze the energetic selectivity of various competing pathways—including dimerization, isomerization, and trimerization—during ethylene oligomerization. Their study focused on metal hydrides (H M–DHKUST-1, where M represents Co, Ni, Ru, Rh, and Pd) supported on defective HKUST-1, providing insights into the mechanistic preferences governing oligomerization reactions. In a separate study in 2019, Müller and colleagues successfully immobilized the Cr-PNP catalyst onto porous silicon carbide (SiC) and activated carbon^33^. They subsequently utilized this immobilized catalyst for oligomerization of ethylene under gaseous phase conditions. In 2019 Fallahi et al.^34^ explored the synthesis of heterogeneous catalysts by immobilizing bis(2-decylsulfanylethyl)amine–CrCl₃ (Cr-SNS-D) onto different solid supports, including commercial TiO₂, Al₂O₃, and magnetic Fe₃O₄@SiO₂ nanoparticles. The resulting catalysts, designated as support@Cr-SNS-D, were systematically analyzed to assess their structural and catalytic properties. In 2020, Shin and colleagues^35^ engineered a series of nickel-based heterogeneous catalysts by functionalizing SBA-15 mesoporous silica with bipyridyl (bpy) ligands, followed by coordination with NiCl₂. The resulting (bpy)Ni(II)-SBA-15 catalysts were systematically assessed for ethylene oligomerization. Their findings revealed that fine-tuning the nickel loading and achieving a uniform distribution of active sites effectively minimized polymeric by-products, thereby improving selectivity toward the targeted oligomers. Furtheremore, in 2020, Li and co-workwers^36^ synthesized a Ni-SiO₂-Al₂O₃ catalyst for ethylene oligomerization, demonstrating its potential for efficient catalytic performance. Additionally, in 2024, Ning et al.^37^ developed three sulfonic acid-functionalized metal-organic frameworks (SA/MIL-101(Cr), UiO-66-NS, and MIL-101(Cr)-NS) and employed them as catalysts for ethylene oligomerization at room temperature, achieving catalytic activity without the need for cocatalysts. Wolny and Chrobok in 2022, highlighted the numerous advantages of supported ionic liquid phases in catalysis^38^. Kong et al. in 2023, introduced a novel class of diphosphinoindole ligands. Chromium catalysts stabilized with these bisphosphine ligands demonstrated significant activity for the tri- and tetramerization of ethylene upon activation with modified methylaluminoxane (MMAO)^39^. By immobilizing ionic liquids on solid supports, they created systems that either exhibit inherent catalytic activity or serve as efficient matrices for active phases, such as enzymes or metal compounds. This approach enhances the stability, reusability, and overall efficiency of the catalysts, making it a promising strategy for various catalytic applications.

**Fig. 1:**
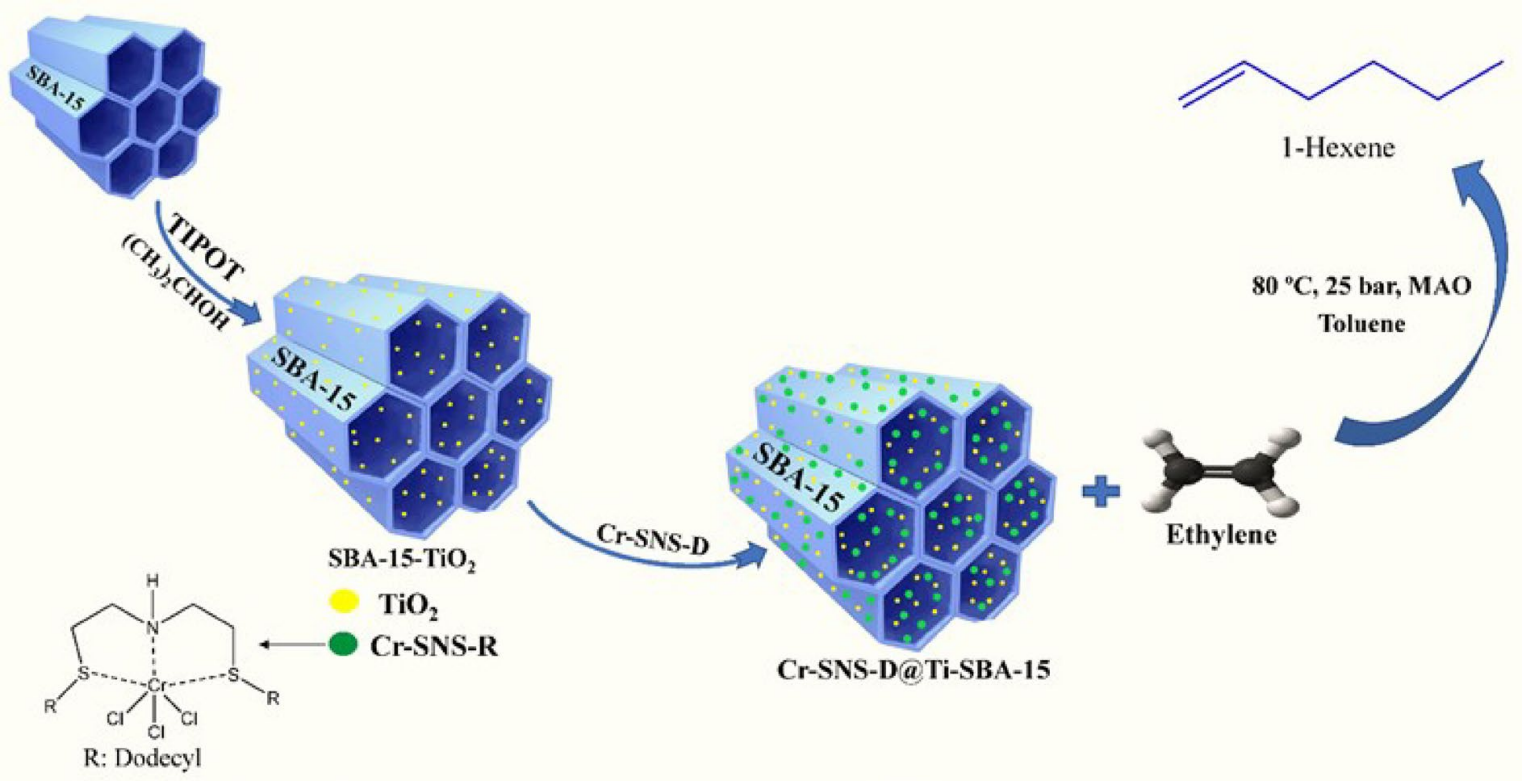
Schematic illustration of the synthesis and catalytic application of Cr-SNS-D@Ti-SBA-15. The SBA-15 mesoporous silica is first functionalized with TiO₂ using TIPOT as the precursor, followed by the immobilization of the Cr-SNS-D complex. The final catalyst, Cr-SNS-D@Ti-SBA-15, is employed in the trimerization of ethylene to selectively produce 1-hexene under optimized reaction conditions (80 °C, 25 bar, MMAO, toluene).

In keeping with our previous studies, two tridentate SNS ligands with butyl (SNS-B) and dodecyl (SNS-D) alkyl groups and their corresponding homogeneous catalysts, Cr-SNS-B and Cr-SNS-D, were synthesized. Then these catalysts were immobilized onto SBA-15 and Ti-SBA-15 ordered mesoporous silica substrates to prepare heterogeneous catalysts. The catalytic activity of the prepared catalysts were compared in homogenous and heterogeneous states. The synthesis pathway and catalytic application of Cr-SNS-D@Ti-SBA-15 are schematically illustrated in Fig. 1. Initially, SBA-15 was functionalized with TiO₂ using TIPOT as the precursor, followed by the immobilization of the Cr-SNS-D complex. The resulting heterogeneous catalyst was then employed for the trimerization of ethylene to selectively produce 1-hexene under optimized reaction conditions.

## Materials and methods

### Materials

Diethyl ether (Et_2_O), toluene, and tetrahydrofuran (THF) were obtained from Merck company and exposed to reflux conditions in benzophenone-sodium under a nitrogen atmosphere to remove water before employing. Tetraethyl orthosilicate (TEOS) and CrCl_3_(THF)_3_ were purchased from Sigma-Aldrich. These chemicals were employed without additional purification. Modified methylaluminoxane (MMAO) (7 Wt.% in toluene), dodecanthiol, bis-(2-chloroethyl) amine hydrochloride, tetraisopropyl orthotitanate (Ti[OCH(CH_3_)_2_]_4_), normal hexane, and ethanol were also obtained from Merck company.

### Preparation of bis(2-alkylsulfanyl-ethyl)amine (SNS-R) ligands, alkyl(R) = dodecyl (D) and Butyl (B)

Tridentate SNS ligands, which include dodecyl and butyl groups, were synthesized according to the procedures outlined in articles^30,34^. First, 4 mL of butanethiol or 8 mL of decanethiol (37 mmol of thiol) introduced into a sodium hydroxide solution (37 mmol, 1.5 g) in 37 mL of ethanol. The obtained solution was maintained under stirring at 0 °C for 20 min. Then, a solution of bis(2-chloroethyl)amine hydrochloride (12 mmol, 2.23 g) in ethanol (25 mL) was added. Stirring was conducted for 4 h at 0 °C and then continued for 16 h at room temperature. The solid phase was then separated, and the liquid phase was removed using a vacuum pump. The resulting viscous liquid was rinsed with anhydrous diethyl ether and anhydrous n-hexane, and the solvent was removed under vacuum. Finally, tridentate SNS ligands with butyl (SNS-B) and dodecyl (SNS-D) groups were obtained with yields of 84% and 70%, respectively.

### Preparation of Cr-SNS-R homogeneous catalysts

First, of SNS-D ligand (0.294 mmol, 139 mg) or SNS-B ligand (0.294 mmol, 73 mg) and chromium trichloride tetrahydrofuran (0.268 mmol, 100 mg) were separately dissolved in dry tetrahydrofuran (10 mL)^40^. The two solutions were then combined, resulting in a rapid transformation of the solution to a blue-green color. The components were stirred for 20 min.

Next, the green solution was placed under a vacuum to remove the solvent. To purify the catalyst, n-hexane (15 mL) or dry diethyl ether was added to the resulting green viscous liquid, which was then refrigerated overnight. Afterward, the solid phase was isolated and then purified by washing with dried diethyl ether (10 mL) more than two times under a vacuum. The entire the reaction and separation process were executed in an argon atmosphere. Finally, the resulting products were named, Cr-SNS-D and Cr-SNS-B.

### Procedure for synthesis of SBA-15 silica support

Following the methodology outlined by Stuckey et al.^41^, a two-dimensional ordered mesoporous silica, SBA-15 was synthesized. HCl (600 mL, 2 M) was added to a 1-liter flask and the flask was placed in a 40 °C oil bath. Pluronic P123 (24 g) was introduced in to the solution subjected to stirring for 2.5 to 3.5 h until the P123 was completely dissolved and yielding a clear solution. Subsequently, tetraethoxy orthosilicate (242.7 mmol, 54.2 mL) was rapidly introduced under an argon-filled environment, and continued stirring the reaction solution for 18 to 22 h to facilitate complete polymerization. The next step involved transferring the resulting material into a balloon and maintaining it in an oil bath within the temperature range of 80 to 90 °C for 24 h. After this period, the mixture was separated by filtration, rinsed with deionized water, and then allowed to dry overnight in an oven set at 60 °C. To remove the P123 template, we refluxed the white solid in ethanol for 50 h. Finally, the resulting white solid underwent calcination at 700 °C with a heating rate of 2 °C/min for 6 h, yielding the SBA-15 substrate.

### Immobilization of Cr-SNS-R catalysts on SBA-15 support

Chromium bis(2-alkylsulfanyl-ethyl)-amine catalyst (Cr-SNS-R) (0.25 mmol, 158 mg Cr-SNS-D or 102 mg Cr-SNS-B) was dissolved in anhydrous toluene (5 mL). Additionally, SBA-15 (0.6 g, which had been pre-treated in a vacuum oven at 100 °C for duration of 12 h) was mixed with dry toluene (10 mL) in a separate 50 mL flask. The catalyst solution was subsequently introduced into the second flask and stirred continuously for 2 h at 40 °C. The obtained green solid was separated, Rinsed three times with anhydrous toluene, and vacuum-dried. All reactions were conducted in an argon environment. The resulting catalysts were named Cr-SNS-D@SBA-15 and Cr-SNS-B@SBA-15.

### Synthesis of nanostructured silica functionalized with titanium (Ti-SBA-15)

To functionalize the SBA-15 with tetraisopropyl orthotitanate (Ti[OCH(CH_3_)_2_]_4_), the following steps were taken: first, tetraisopropyl orthotitanate (0.608 mmol, 0.18 mL) and 2-propanol (47.03 mmol, 3.6 mL) were added to the reaction vessel in a nitrogen environment. Next, SBA-15 (0.6 g) was incorporated into the reaction solution and subjected to stirring for 90 min under an argon atmosphere. After that, deionized water (1.8 mL) was added slowly and continuously to the reaction media while stirring for an additional 30 min under ambient conditions. The resultant solid was subsequently filtered and isolated. It was rinsed several times with hot ethanol and ultimately placed in an oven set at 110 °C for 20 h to achieve dehydration. Lastly, the solid was subjected to calcination at 700 °C for 7 h, heating at a rate of 10 °C/min, to eliminate residual organic compounds.

### Immobilization of chromium bis(2-dodecylsulfanyl-ethyl)-amine catalyst (Cr-SNS-R) on Ti-SBA-15

Chromium bis(2-alkylsulfanyl-ethyl)-amine catalyst (0.25 mmol, 158 mg Cr-SNS-D or 102 mg Cr-SNS-B) was dissolved in anhydrous toluene (5 mL). In a separate flask, Ti-SBA-15 (0.6 g) which had been vacuum-dried for 12 h at 100 ^o^C before the reaction was mixed with of dry toluene (10 mL). The catalyst solution was then introduced into the second flask. The reaction solution was continuously mixed at 40 °C over a period of 2 h. Afterward, the green solid was separated and rinsed three times with anhydrous toluene. The catalyst was then subjected to vacuum drying.

### General agenda for the ethylene trimerization

Ethylene trimerization was conducted in a 200 mL stainless-steel reactor equipped with a mechanical agitator. The reactor was first dried and purged with argon gas at 100 °C for 2 h. Once the reactor reached the target temperature of 80 °C, 40 mL of anhydrous toluene was injected, followed by the addition of a modified methyl aluminoxane (MMAO) co-catalyst. Ethylene gas was continuously flowed through the system, and after 20 min, the catalyst (2.3 µmol) was introduced, and the ethylene pressure was set to 25 bar. The reaction proceeded for 30 min, after which the reactor was cooled to room temperature. The liquid phase was then collected for analysis by gas chromatography (GC). The trimerization was performed under the optimized conditions identified in our previous research (MMAO co-catalyst with Al/Cr = 700/1) to evaluate the performance of the catalysts: Cr-SNS-D, Cr-SNS-B, SBA-15, and Ti-SBA-15 ^5^. The trimerization product was then analyzed using GC to determine the composition, including the main product 1-hexene and other side products such as polyethylene, in the presence of MMAO co-catalyst.

### Instrument

The samples were investigated using a Bruker-Vector 22 FT-IR spectrometer to obtain FT-IR spectra in the range of 400 to 4000 cm^-1^. A Shimadzu UV-1650PC UV-Vis spectrophotometer, with Barium sulfate as the standard sample, was used for UV spectroscopy. The ligands were analyzed using a Brucker Advance DPX-250 MHZ apparatus to assign the^1^H NMR and^13^C NMR spectra. The analysis was performed at ambient temperature in CDCl_3_ solvent. A Vario EL III CHNS elemental analyzer was used to determine the elemental composition. After trimerization, the resulting mixture was evaluated using gas chromatography (GC) Varian CP-3800 model with a flame ionization detector (FID). The specifications are as follows: column length of 60 m, column diameter of 0.25 mm, and film thickness of 1 micrometer. The injector temperature is set to 250 °C, with an injection volume of 0.1 µL. The carrier gas used is nitrogen, and the column pressure is maintained at 50 psi. Nitrogen adsorption-desorption isotherms were measured at 77 K using an OMNISORP 100CX VER 1G adsorption apparatus. The samples were analyzed using a NETZSCH STA 409 PC/PG thermogravimetric analyzer, scanning from 25 to 800 ˚C at a rate of 10 ˚C/min. The morphology of the supported catalysts was characterized using FEI ESEM QUANA 200 scanning electron microscopy (SEM) and elemental mapping with a TESCAN TS 5130 VEGA instrument. The Ti concentration was determined via inductively coupled plasma atomic emission spectroscopy (ICP-OES 730-ES, Varian).

## Results and analysis

### Characterization of the ligands and catalysts

**Fig. 2:**
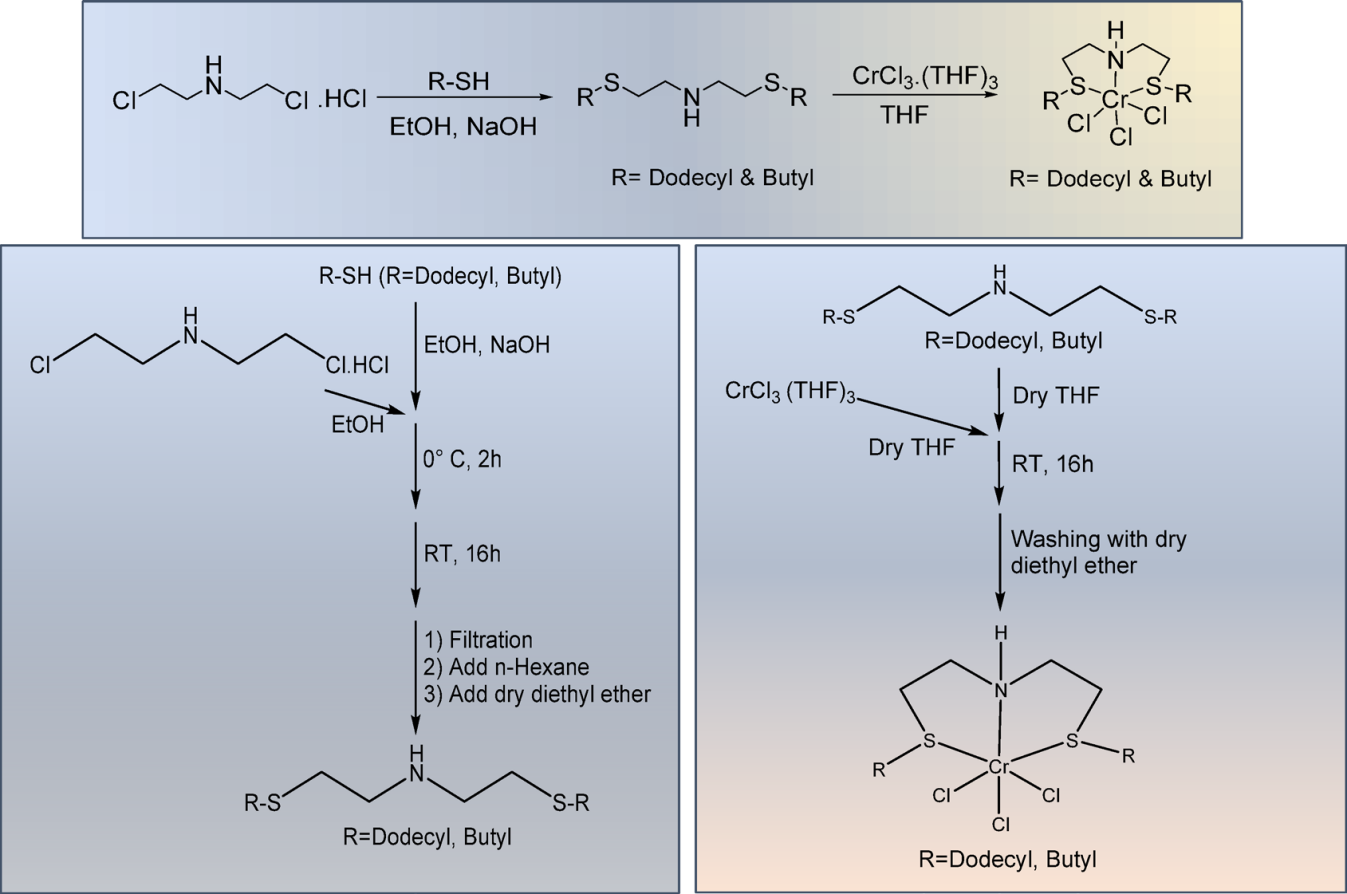
The synthesis pathway for producing Cr-SNS-R catalysts.

To characterize the ligands and catalysts, we employed various techniques. The synthesis mechanism, chemical structure,^1^H NMR and^13^C NMR spectra of the produced SNS-D are detailed in the supporting information (Figures S1-S4), while those for SNS-B are provided in Figures S5-S7. Figure S8 presents the FT-IR spectra of the fabricated SNS-D and SNS-B ligands. Additionally, the CHNS analysis data for these ligands are presented in Table S1, confirming the successful synthesis of SNS-D and SNS-B ligands. The general pathway for producing the Cr-SNS-R catalyst is outlined in Figs. 2 and S9. Furthermore, the FT-IR spectrum of the Cr-SNS-R complexes is shown in Figure S10, and their CHNS elemental analysis is presented in Table S2.

### Characterization of Ti-SBA-15 support

**Fig. 3:**
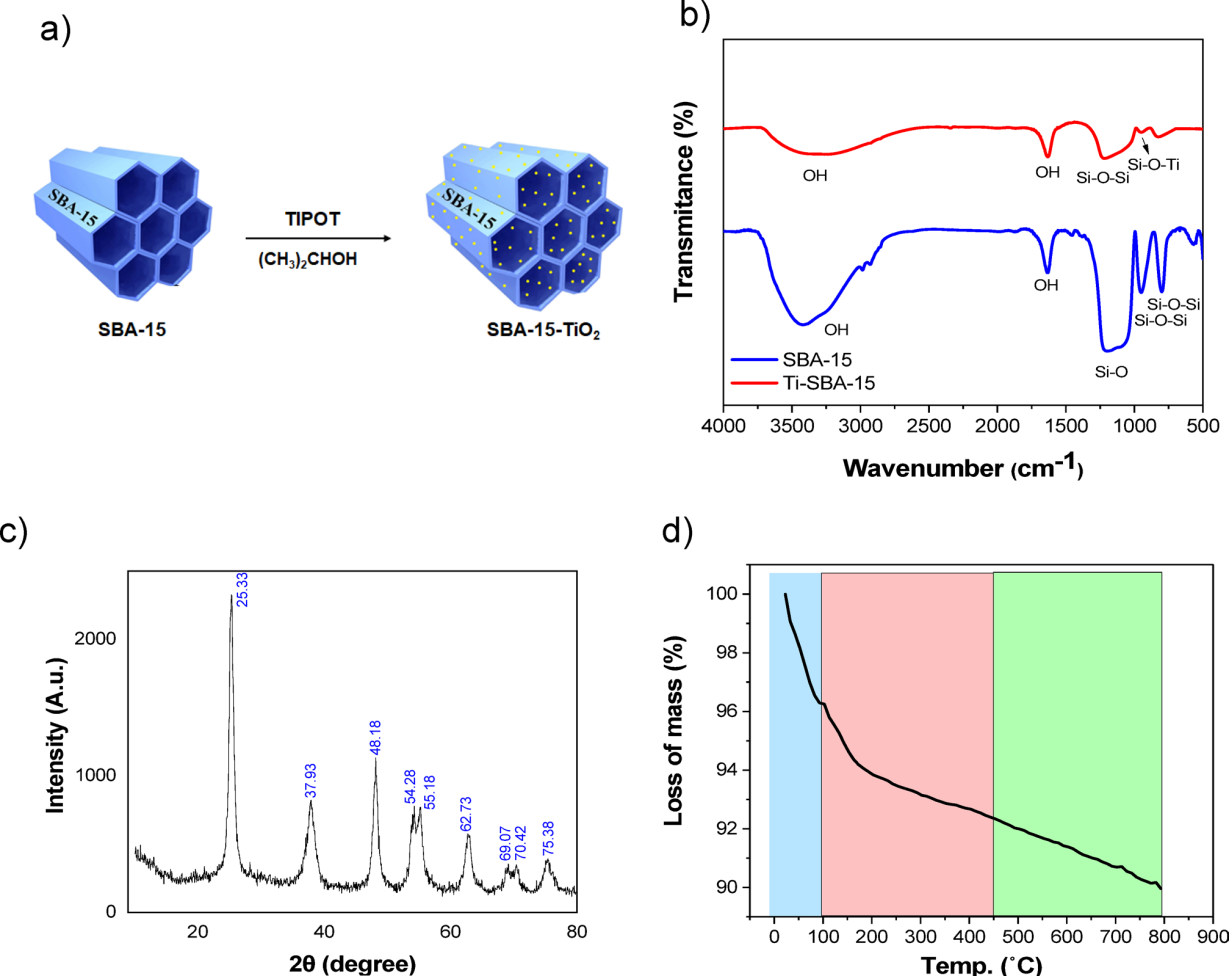
(**a**) Schematic illustration for preparation of Ti-SBA-15. (**b**) FT-IR spectrum of SBA-15 and Ti-SBA-15 supports. (**c**) X-ray diffraction pattern of Ti-SBA-15. (**d**) Thermal gravimetric analysis (TGA) diagram of Ti-SBA-15 sample in a nitrogen atmosphere with a heating rate of 10 °C/min.

SBA-15 mesoporous silica formulated in agreement with the Stuckey’s method. To determine the structural characteristics of SBA-15, various techniques including FT-IR (Figure S11), nitrogen absorption-desorption (Figure S12), and X-ray diffraction (XRD) were employed (Figure S13). The ICP device was used to measure the weight% of titanium in the prepared substrate, Ti-SBA-15. Following the method explained in Sect. 2.6, Ti-SBA-15 was prepared (Fig. 3a) and actual weight% of titanium in the sample was determined by the ICP device. It was found that 19.46% by weight of titanium was present on the substrate. Figure 3b illustrates the FT-IR spectroscopy of calcined silica mesopore Ti-SBA-15. In the 3450 cm^-1^ region, there is a broad band that corresponds to stretching vibrations. The absorption peak at around 1640 cm^-1^ can be explained by the bending vibrations of the surface O-H groups. The absorption band at 1080 cm^-1^ is related to the asymmetric stretching vibrations of Si-O-Si linkages. The symmetric stretching vibrations of Si-O-Si groups are detected in the range of 795 –790 cm^-1^. The presence of Ti in the sample is indicated by the peak at 1960 cm^-1^, which is associated with Ti-O-Si. This result indicates that the Ti atoms are chemically bonded to the silica. Wide angle X-ray diffraction analysis was conducted to examine the crystalline phases of titanium dioxide particles stabilized on the SBA-15 mesoporous silica substrate. TiO₂ commonly exists in three polymorphic forms: anatase, rutile, and brookite, each with distinct properties and applications. The XRD patterns revealed that the TiO₂ particles deposited on the SBA-15 substrate predominantly exhibit a combination of rutile and anatase phases. This phase distribution arises from the stabilization effects imparted by the mesoporous silica surface, which can influence the phase transition dynamics of TiO₂^42^. The rutile phase tends to favor the formation of the anatase phase. Analysis of the diffraction pattern (Fig. 3c) confirms the formation of high-purity anatase with minimal rutile content. The characteristic peaks observed at 2θ ≈ 25.3° (anatase) further validate the co-existence of these two phases. The absence of additional peaks from other crystalline forms, such as brookite, suggests that the TiO₂ particles are predominantly in anatase and rutile forms, with anatase being the dominant phase. The thermal endurance of the Ti-SBA-15 substrate was investigated by thermal gravimetric analysis. The overall weight loss is shown in the Fig. 3d. The initial weight loss, which amounts to 3.67%, occurs up to a temperature of approximately 100 ˚C. This decrease is clearly depicted in the graph and corresponds to the elimination of water molecules that are adhered to the surface. The second weight loss, totaling 2.19%, occurs up to 500 ˚C and is due to the removal of the remaining P123 surfactant found within the channels of SBA-15. Finally, there is a final weight loss 4.09% observed after reaching 500 ˚C, which can be attributed to the phase change of the substrates (anatase to rutile) and the conversion to the crystalline form of the substrate in question.

### Characterization of the heterogeneous catalysts

**Fig. 4:**
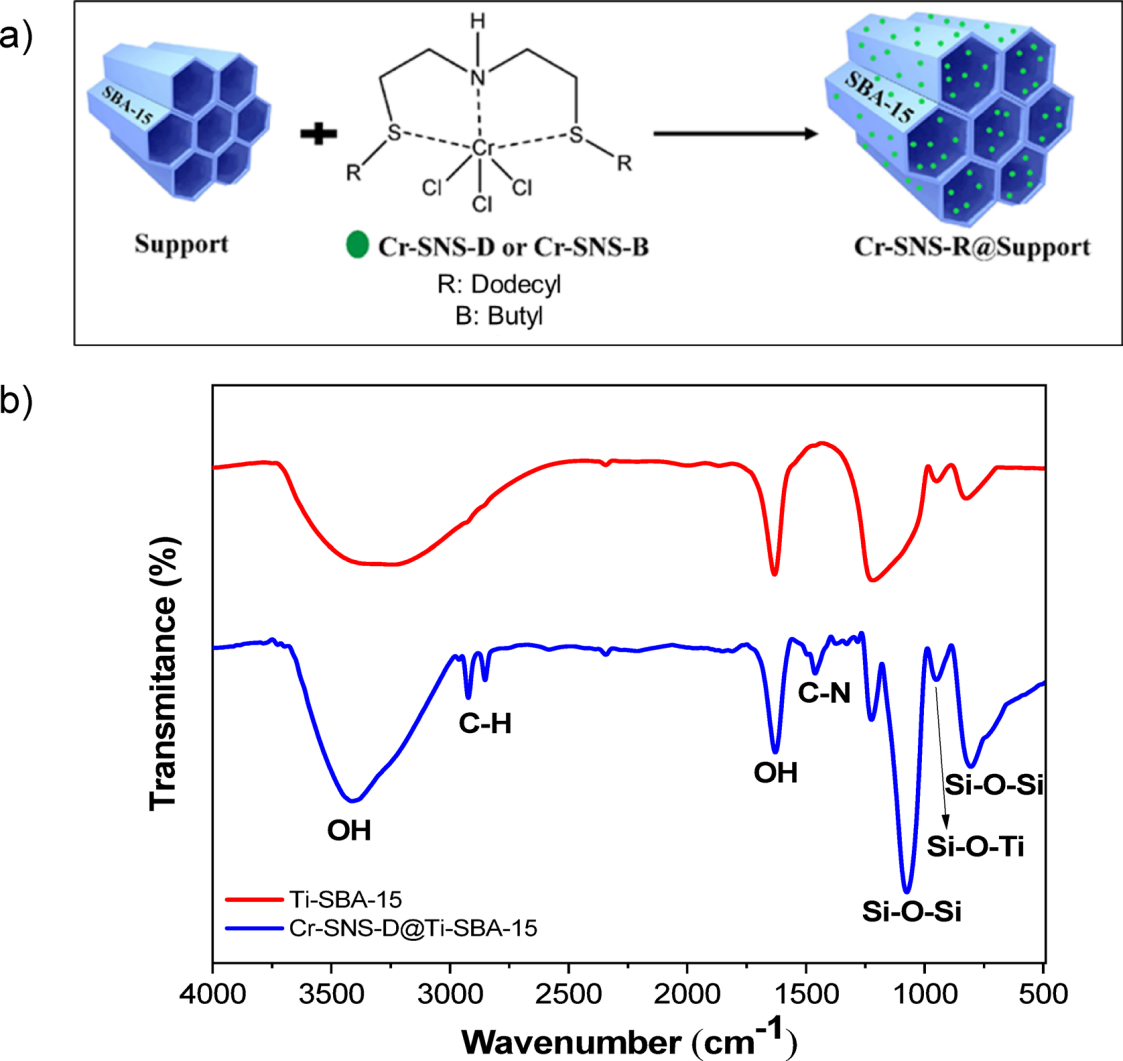
(**a**) Schematic for the production of heterogeneous Cr-SNS-R@Ti-SBA-15 catalysts. (**b**) FT-IR spectra to compare the synthesized Ti-SBA-15 and Cr-SNS-D@Ti-SBA-15.

The Cr-SNS-R complex was prepared using the method outlined in the literature^30^. The complex was subsequently immobilized on various supports, including SBA-15 and Ti-SBA-15, resulting in the heterogeneous catalysts referred to as Cr-SNS-R@Support. Figure 4a outlines the general approach for preparing chromium-supported catalysts. The provided catalysts were thoroughly characterized employing techniques such as FT-IR spectroscopy (Figure S14) for Cr-SNS-B@SBA15 and Cr-SNS-D@SBA-15, diffuse reflectance UV-visible spectroscopy (Figure S15), and thermal gravimetric analysis (TGA) (Figures S16, S17) for Cr-SNS-D@SBA-15 and Cr-SNS-B@SBA-15, respectively.

### Characterization of Cr-SNS-R@Ti-SBA-15 catalyst

Initially, the modified mesoporous silica Ti-SBA-15 was subjected to calcination at 700 °C to minimize the amount of the surface hydroxyl groups. Subsequently, the complexes (Cr-SNS-R) with dodecyl (Cr-SNS-D) and butyl (Cr-SNS-B) alkyl groups were effectively stabilized onto a titanate functionalized silica substrate to prepare Cr-SNS-R@Ti-SBA-15 catalysts. The synthesized catalysts were evaluated through infrared spectroscopic analysis, porosimetry, scanning electron microscopy, and elemental mapping. Figure 4b shows the FT-IR spectroscopy of the Cr-SNS-D@Ti-SBA-15 catalyst. Through comparison the FT-IR spectra of the developed SNS chromium-based catalysts with dodecyl side branches with titanate-functionalized SBA-15 (Cr-SNS-D@Ti-SBA-15) and titanate-functionalized SBA-15 (Ti-SBA-15), can be inferred that the chromium catalyst has been successfully stabilized on Ti-SBA-15. There is a wide band observed in the region of 3450 cm^− 1^, which is associated with the stretching vibrations of O-H bonds, typically found in hydroxyl groups. In addition, there is an absorption peak at about 1640 cm^− 1^, which is related to the bending vibrations of the surface O-H groups. The peaks at 2920 and 2850 cm^− 1^ correspond to the symmetric and asymmetric C-H stretching vibrations, confirming the presence of the substrate (Ti-SBA-15) on the complex (Cr-SNS-R). The absorption peak at approximately 1080 cm^− 1^ is related to the asymmetric stretching vibrations of Si-O-Si groups. Furthermore, the symmetric stretching vibrations of Si-O-Si groups are detected in the 790–795 cm^− 1^ range. Finally, the peak at 960 cm^− 1^ is associated with Ti-O-Si^43,44^.

**Fig. 5:**
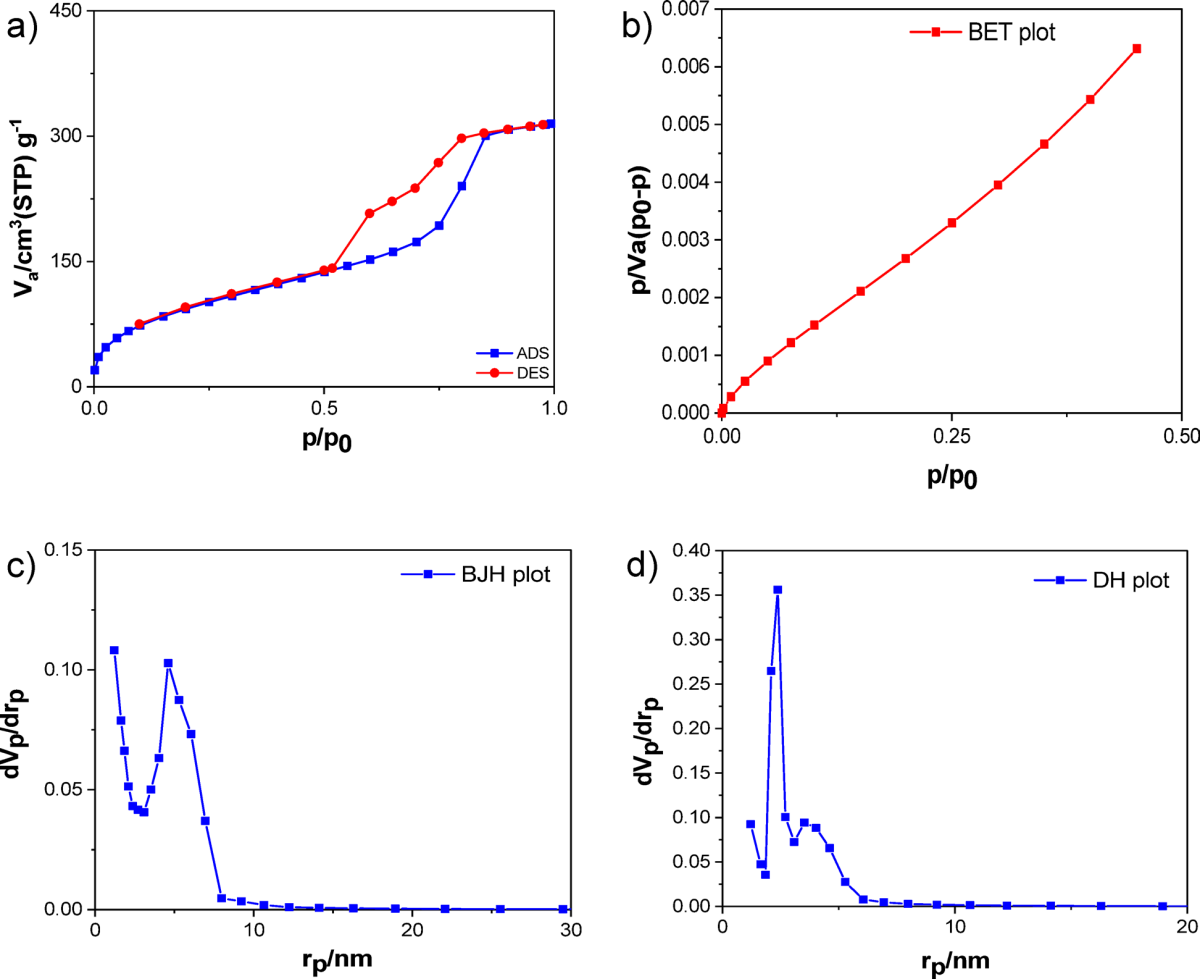
(**a**) Nitrogen adsorption-desorption isotherm of mesoporous silica functionalized with titanium (Cr-SNS-D@Ti-SBA-15). (**b**) BET plot for Cr-SNS-D@Ti-SBA-15. (**c**) Barrett-Joyner-Halenda (BJH) pore size distribution curve derived from the desorption branch. (**d**) Differential height (DH) curve for Cr-SNS-D@Ti-SBA-15.

Figure 5a displays the nitrogen adsorption-desorption analysis at 77 K for the Cr-SNS-D catalyst supported on a Ti-SBA-15. The graph illustrates a type IV isotherm with an absorption-desorption loop and a gradual slope in the relative pressure (*P*/*P*_*0*_) range of 0.5 to 0.9. This suggests a high degree of order within the synthesized nanostructure. Additionally, the sample displays a hysteresis curve of the first type H1, suggesting the presence of fully uniform and cylindrical cavities. Based on the BET calculations (Fig. 5b), the effective surface area of this compound is 352 m^2^/g, and the total volume of voids is 0.48 cm^3^/g. By stabilizing the Cr-SNS-D catalyst within the titanium-functionalized mesoporous channels, the cavity space is reduced, leading to a reduction in both the cavity capacity and the specific surface area. However, the regular structure of the holes in the initial mesoporous composition remains intact even after the surface modification with the catalyst. The results of the BJH calculations also indicate that the synthesized structure possesses regular holes with a consistent arrangement and an average diameter of 52.5 nm (Fig. 5c). Moreover, DH curve investigated for further confirming the regular mesoporous nanostructure (Fig. 5d).

The nitrogen adsorption-desorption isotherms for SBA-15, Cr-SNS-D@SBA-15, Cr-SNS-B@SBA-15, Cr-SNS-D@Ti-SBA-15, and Cr-SNS-B@Ti-SBA-15 catalysts were analyzed, and the resulting surface properties are summarized in Table 1. The data reveal a notable decrease in the BET surface area (S_BET_) and total pore volume (V_p_) of SBA-15 following the immobilization of chromium-based catalysts, confirming the successful incorporation of Cr-SNS-R complexes within the mesoporous framework. Specifically, the surface area of pure SBA-15 (1042 m^2^/g) decreases to 763 m^2^/g for Cr-SNS-D@SBA-15 and further to 547 m^2^/g for Cr-SNS-B@SBA-15, while the pore volume drops from 1.20 cm^3^/g to 0.98 cm^3^/g and 0.80 cm^3^/g, respectively. This reduction is more pronounced with Cr-SNS-B, which features a shorter butyl chain, compared to Cr-SNS-D with a longer dodecyl chain. The observed trend likely arises from the greater penetration of the shorter-chain Cr-SNS-B into the mesopores, leading to more significant occlusion of the pore structure, as the hydrophilic nature of the silica surface may limit the penetration of the longer, more hydrophobic dodecyl chains of Cr-SNS-D. These structural modifications have a direct impact on catalytic activity, selectivity, and stability. The decrease in surface area and pore volume suggests a trade-off between active site accessibility and dispersion. While the immobilization of Cr complexes within SBA-15 enhances their stability by preventing aggregation, excessive pore occlusion—especially in Cr-SNS-B@SBA-15—can limit the diffusion of reactants, potentially reducing overall catalytic activity. However, the better confinement of active sites in Cr-SNS-B@SBA-15 may also contribute to improved selectivity by minimizing unwanted secondary reactions leading to by-products. In terms of stability, the immobilization of Cr complexes within the mesoporous structure of SBA-15 provides enhanced resistance to leaching and deactivation compared to homogeneous catalysts. The stronger interaction between Cr-SNS-B and the support, due to deeper penetration into the pores, likely contributes to increased catalyst lifetime under reaction conditions. However, an optimal balance is required, as excessive confinement may hinder reactant accessibility and reduce catalytic efficiency. Thus, the observed differences in textural properties between Cr-SNS-D@SBA-15 and Cr-SNS-B@SBA-15 highlight the crucial role of ligand structure in determining catalyst performance, with implications for activity, selectivity, and long-term stability.

**Table 1:** Surface properties of SBA-15, Cr-SNS-D@SBA-15, Cr-SNS-B@SBA-15, Cr-SNS-D@Ti-SBA-15, Cr-SNS-B@Ti-SBA-15.

Entry	Sample	S_BET_ (m^2^/g)	V_*p*_ (cm^3^/g)	D_*p*_ (nm)
1	SBA-15	1042	1.20	4.63
2	Cr-SNS-D@SBA-15	763	0.98	5.15
3	Cr-SNS-B@SBA-15	547	0.80	5.85
4	Cr-SNS-D@Ti-SBA-15	352	0.48	5.52
5	Cr-SNS-B@Ti-SBA-15	230	0.22	3.86

S_BET_: Specific surface area, V_P_: Total pore volume, D_P_: Pore diameter.

**Fig. 6:**
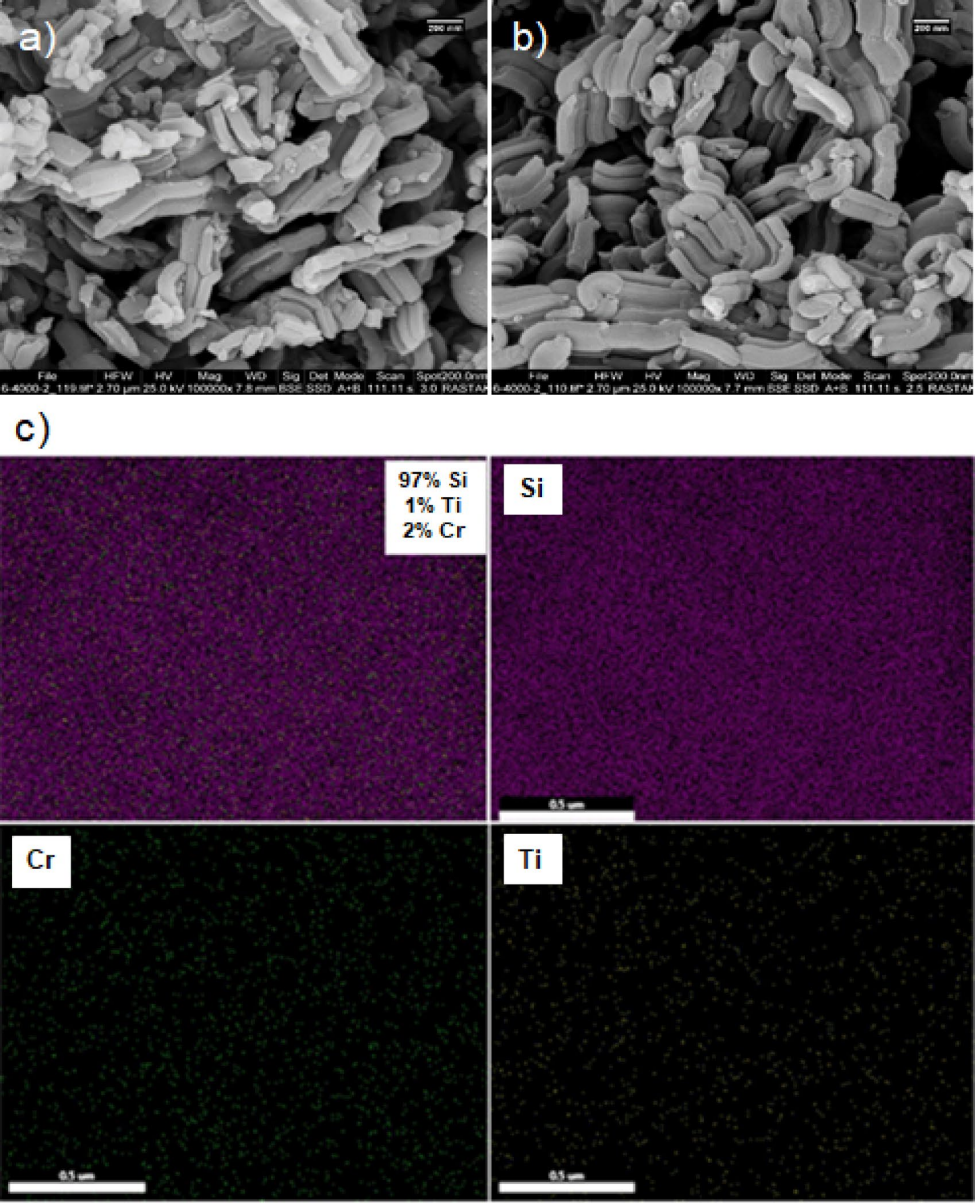
(**a**) Scanning electron microscopy (SEM) image of Cr-SNS-D@Ti-SBA-15. (**b**) SEM image of pure SBA-15. (**c**) Elemental mapping images of Cr-SNS-D@Ti-SBA-15 obtained via energy-dispersive X-ray spectroscopy (EDS), illustrating the uniform distribution of chromium (Cr), titanium (Ti) and silicon (Si).

Figure 6a and b present scanning electron microscopy (SEM) images that compare the morphology of the Cr-SNS-D@Ti-SBA-15 catalyst and pure SBA-15. The SEM images show that the rod-like structure of the catalyst remains intact after its attachment to the Ti-SBA-15 substrate, confirming the stability of the mesoporous framework. Figure 6c displays elemental mapping images of Cr-SNS-D@Ti-SBA-15 obtained via energy-dispersive X-ray spectroscopy (EDS), clearly illustrating the uniform distribution of chromium atoms across the mesoporous silica (SBA-15) substrate that has been functionalized with titanium. This uniform dispersion is essential for improving the catalytic activity of the Cr-SNS-D@Ti-SBA-15 system.

After fully characterizing the catalysts (Cr-SNS-D, Cr-SNS-B), SBA-15, and Ti-SBA-15, the we studied the trimerization of ethylene.

### Ethylene trimerization

The selectivity and activity of Cr-SNS-D and Cr-SNS-B homogeneous catalysts were examined in the ethylene trimerization process. The findings indicated that the homogeneous catalysts Cr-SNS-D and Cr-SNS-B had yields of β-hydrogen and 53,344 g 1-C₆ g Cr^− 1^.h^− 1^, respectively. Table 2 reports a selectivity of 99.99%. The Cr-SNS catalysts demonstrated high selectivity and activity, making them cost-effective for practical and industrial use due to the affordable price of the ligands. The superior activity of Cr-SNS-D compared to Cr-SNS-B can be ascribed to its longer chain length, indicating better solubility of the Cr-SNS-D catalyst in toluene solvent. Moreover, reduced steric hindrance facilitates the approach of ethylene to the active catalytic center. According to the bridges mechanism, ethylene replacement and the formation of 1-hexene are more readily achieved. The role of the MMAO cocatalyst is to eliminate water and oxygen contamination from the reaction environment. In addition to investigating the effect of heterogeneous catalysts on the ethylene trimerization process, Cr-SNS-D and Cr-SNS-B were immobilized on conventional mesoporous silica substrates, SBA-15 and Ti-SBA-15. Mesoporous silica is commonly utilized as a catalyst substrate owing to its broad surface area and adjustable pore dimensions. However, the presence of numerous silanol groups on its surface can lead to inactivity. Therefore, to prepare catalysts with high activity in ethylene trimerization, these materials were first calcined at 700 ºC under argon flow post-synthesis to minimize the number of surface hydroxyls. Then, chromium catalysts were immobilized on them, resulting in stabilized chromium species on the surface. The results obtained from ethylene trimerization using heterogeneous catalysts are presented in Table 2, Entries 3–5. Similar to homogeneous catalysts, Cr-SNS-D catalysts showed enhanced activity than Cr-SNS-B in the heterogeneous system. Despite the promising performance of the Cr-SNS-D@SBA-15 heterogeneous catalyst in ethylene trimerization, its activity reached only about one-third of that of the homogeneous catalyst under identical reaction environment. Consequently, the production of 1-hexene was lower, amounting to 19,394 g 1-C₆ g Cr^− 1^.h^− 1^.The outcomes from the ethylene trimerization reaction using heterogeneous catalysts Cr-SNS-B@SBA-15 and Cr-SNS-D@SBA-15 under optimal conditions are provided in Table 2, Entries 3 and 4.

**Table 2:** Ethylene trimerization for heterogeneous catalysts.

Entry	Catalyst	1-Hexene (mL)	Selectivity 1-C₆ (wt%)	Activity (g 1-C₆ g Cr^− 1^.h^− 1^)	PE (g)
1	Cr-SNS-B	4.74	99.9	53,344	0.04
2	Cr-SNS-D	5.40	99.9	60,772	0.05
3	Cr-SNS-B@SBA-15	1.18	99.6	12,471	0.70
4	Cr-SNS-D@SBA-15	1.70	99.7	19,394	0.40
5	Ti-SBA-15	0	0	0	0
6^[a]^	Cr-SNS-D@Ti-SBA-15	0	0	0	0
7	Cr-SNS-D@Ti-SBA-15	3.12	99.7	35,115	0

Reaction Conditions: catalyst (2.3 µmol Cr), 30 min, 80 ºC, 25 bar, 40 mL toluene, Cr: Al (1:700) [a]: without MMAO.

TiO_2_ was used for the first time to modify SBA-15 in the ethylene trimerization process, for the purpose of investigate its impact on the catalyst activity of the substrate. The activity of the heterogeneous catalyst with the butyl group was found to be lower than that of dodecyl. To continue the work on the Cr-SNS-D catalyst, it was stabilized on silica mesoporous modified with TiO_2_ (Ti-SBA-15), leading to the identification of the catalyst Cr-SNS-D@Ti-SBA-15. When comparing the activity of Cr-SNS-D@SBA-15 and Cr-SNS-D@Ti-SBA-15 as heterogeneous catalysts, it was observed that the titanium-modified substrate increased the catalytic activity by about 1.5 times. This increase in activity can be assigned to the reduction of surface hydroxyl functionalities following the modification with titanium. This reduction in hydroxyl groups leads to a decrease in their interaction with chromium species and MMAO cocatalyst during the ethylene trimerization process. Additionally, the electronic effect of the substrate can also enhance the catalytic performance and boost the production of 1-hexene. The use of the titanium-based substrate not only increased the activity but also significantly reduced the production of polyethylene as a by-product (Table 2, Entry 7). To verify that the chromium catalytic species were responsible for the ethylene trimerization reaction, a parallel experiment was conducted using Ti-SBA-15 and MMAO as the catalyst, excluding the chromium species (see Table 2, Entry 5), and no product was formed in the absence of chromium species. Furthermore, the ethylene trimerization reaction was carried out using the Cr-SNS-D@Ti-SBA-15 catalyst without MMAO cocatalyst (Table 2, Entry 6). Based on the obtained information, it was found that the titanium species alone cannot act as a catalyst or cocatalyst, but it can improve the catalytic properties. Overall, the investigations revealed that the Cr-SNS-D@Ti-SBA-15 catalyst is the most efficient heterogeneous catalyst in the trimerization process. As shown in Table 3, the comparative investigation of chromium-based catalysts for ethylene trimerization and 1-hexene production reveals significant variations in catalytic performance based on the support type, cocatalyst, and reaction conditions. Notably, catalysts supported on Ti-SBA-15, Fe₃O₄@SiO₂, Al₂O₃, and TiO₂ exhibit exceptionally high selectivity (~ 99.7–99.8%) toward 1-hexene, highlighting the crucial role of support interactions in stabilizing active sites and suppressing side reactions. In contrast, silica-supported Cr-based catalysts (e.g., Cr/SNS, Cr[N(SiMe₃)₂]₃) demonstrate moderate selectivity (74.2–97.3%), while Cr/Ti/SiO₂ with TEAl modification exhibits the lowest selectivity (~ 77.8%), suggesting that the incorporation of titanium in silica frameworks may influence oligomerization pathways. This study highlights the critical influence of support modification on catalytic behavior, demonstrating how tailored electronic interactions between the active sites and the support can optimize performance. The findings provide a valuable framework for designing more efficient chromium-based catalysts, bridging the gap between homogeneous and heterogeneous systems for improved 1-hexene production. This innovative approach distinguishes our work by focusing on the optimization of support interactions, leading to enhanced selectivity and performance in the trimerization process compared to previous chromium-based catalyst systems.

**Table 3:** Comparison of chromium-based catalysts for 1-hexene production.

Catalyst Composition	Support Material	Reaction Conditions	Selectivity to 1-Hexene (%)	Yield (if reported)	Reference
Cr-SNS-D@Ti-SBA-15	Ti-SBA-15	80 °C, 25 bar, MMAO (Al/Cr = 700/1).	99.7%	Not reported	This study
Cr[N(SiMe₃)₂]₃ with isobutylalumoxane	Silica (600 °C calcined)	120 °C and 0.7 MPa.	74.2%	50 g in 2 h	^45^
Cr/SNS	SiO_2_	90 °C, 30 and 40 bar, 480 equivs of MMAO-3 A	97.3%	Not reported	^46^
Cr/Ti/SiO_2_	SiO₂ modified with TiO₂	80 °C, 20 bar, TEAl modification	77.8%	Not reported	^47^
Fe_3_O_4_@SiO_2_@Cr-SNS‐D	Fe_3_O_4_@SiO_2_	80 °C and 25 bar, MAO (Al/Cr = 700)	99.7%	Not reported	^34^
Al_2_O_3_@Cr-SNS‐D	Al_2_O_3_	80 °C, 25 bar, MAO (Al/Cr = 700)	99.8%	Not reported	^34^
TiO_2_@Cr-SNS‐D	TiO_2_	80 °C, 25 bar, MAO (Al/Cr = 700)	99.8%	Not reported	^34^
TiO_2_@Cr-SNS‐D	TiO_2_	80 °C, 25 bar, without MAO	-	Not reported	^34^

### Mechanism of ethylene trimerization using the catalysts supported on silica modified with titanium

**Fig. 7:**
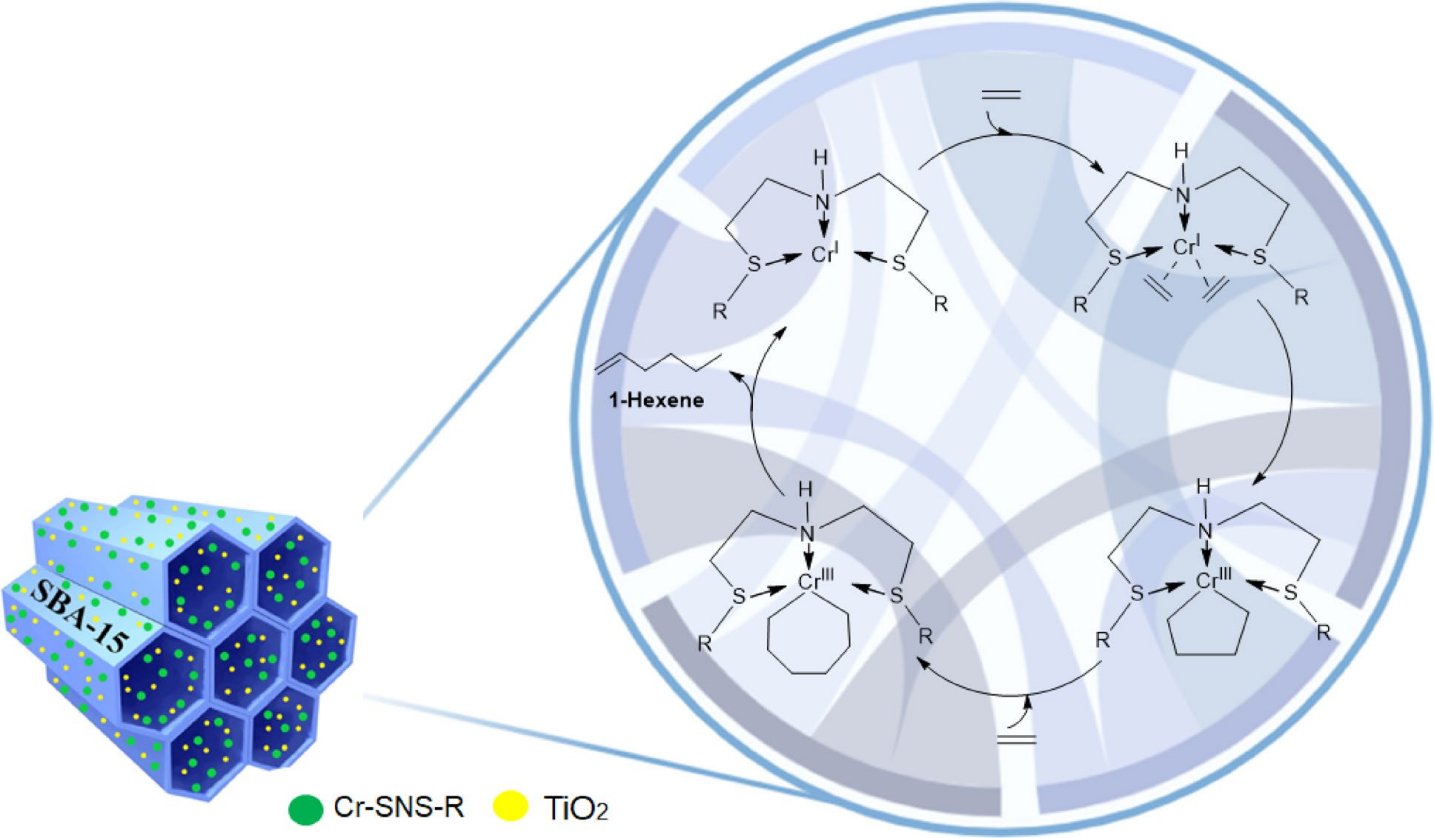
Mechanism for trimerizing ethylene with chromium-based catalysts.

The ethylene trimerization mechanism utilizing titanium-modified silica-supported chromium catalysts follows a well-defined catalytic cycle, as illustrated in Fig. 7. The catalyst consists of a Cr-SNS-R complex immobilized on SBA-15 mesoporous silica, which has been modified with titanium dioxide (TiO₂). The catalytic process begins with the activation of the chromium center in the presence of a co-catalyst, such as modified methylaluminoxane (MMAO), generating an active Cr(III) species with vacant coordination sites for ethylene coordination. In the first step, ethylene molecules coordinate to the Cr(III) center via π-complexation, followed by migratory insertion into the Cr–C bond, leading to the formation of a metallacyclopentane intermediate. Subsequently, a second ethylene molecule inserts into the metallacyclopentane ring, yielding a seven-membered metallacycloheptane intermediate. This step is critical for controlling the selectivity of the reaction toward 1-hexene production^47^ The final stage involves β-hydrogen elimination from the metallacycloheptane intermediate, resulting in the directed synthesis of 1-hexene and regenerating the active Cr(III) species for the next catalytic cycle. The titanium modification of the SBA-15 support plays a crucial role in enhancing the performance of the catalyst. The presence of TiO₂ reduces surface hydroxyl groups, preventing undesired catalyst deactivation and increasing the catalyst’s stability under reaction conditions. Furthermore, titanium modification facilitates better ethylene adsorption onto the silica surface, improving the accessibility of ethylene to the active chromium sites and promoting more efficient polymerization. The mesoporous structure of SBA-15 provides a high surface area and well-defined pore channels, which allow for better diffusion of reactants and the creation of more active sites for the polymerization process. Additionally, TiO₂ enhances electronic interactions between the titanium-modified silica and the Cr active sites, further increasing the catalytic activity. As a result, the Ti-modified silica support minimizes the formation of by-products, such as polyethylene, ensuring high selectivity toward 1-hexene production. Thus, the Ti-SBA-15 catalyst is active for polymerization because the titanium modification improves the structural stability of the catalyst, promotes better ethylene coordination, and increases the overall catalytic efficiency, contributing to both enhanced activity and selectivity in the ethylene trimerization process.

## Conclusion

In this study, we investigated the ethylene trimerization activity and selectivity of both homogeneous and heterogeneous chromium-based catalysts. The homogeneous catalysts, Cr-SNS-D and Cr-SNS-B, demonstrated high selectivity (99.9%) with activities of 60,772 and 53,344 g 1-C₆ g Cr⁻¹ h⁻¹, respectively, under optimal reaction conditions. However, when immobilized on titanium-modified SBA-15 supports, the heterogeneous catalysts exhibited slightly reduced activity, with Cr-SNS-D@SBA-15 and Cr-SNS-B@SBA-15 showing activities of 19,394 and 12,471 g 1-C₆ g Cr⁻¹ h⁻¹, respectively. Notably, the titanium-modified catalyst, Cr-SNS-D@Ti-SBA-15, showed a substantial increase in activity—approximately 1.5 times higher than its non-modified counterpart, reaching 35,115 g 1-C₆ g Cr⁻¹ h⁻¹. This improvement in catalytic performance was accompanied by a significant reduction in polyethylene by-product formation, highlighting the effectiveness of titanium modification in enhancing both catalytic activity and selectivity. These results underscore the importance of support modification in heterogeneous catalysis, where Ti-SBA-15 provides better ethylene adsorption and electronic interaction with the chromium sites, leading to improved catalyst efficiency. The findings suggest that titanium modification offers a promising strategy for improving the performance of chromium-based catalysts in ethylene trimerization, providing high selectivity towards 1-hexene and minimizing undesirable by-products. Future work could explore further optimization of the Ti-SBA-15 support and other modifications to enhance catalyst reusability and long-term stability.

## Electronic supplementary material

Below is the link to the electronic supplementary material.

Supplementary Material 1

## Data Availability

The datasets used and/or analysed during the current study available from the corresponding author on reasonable request.
