# Supplementary material for: Selective trimerization of ethylene using chromium catalysts complexed with tridentate ligands supported on titanium modified silica
Source: Sci Rep. 2025 May 25;15:18193. doi: 10.1038/s41598-025-02844-9 (PMC12104422; doi:10.1038/s41598-025-02844-9)
Supplement: Supplementary file 1 — Supplementary Material 1 [file 41598_2025_2844_MOESM1_ESM.docx]

**Supporting information**

**for**

**Selective Trimerization of Ethylene Using Chromium Catalysts Complexed with Tridentate Ligands Supported on Titanium Modified Silica**

Younes Habibi^a^, Mohamadreza Marefat^b^, Sajjad Gharajedaghi^a^, Masoumeh Mohamadhoseini ^a^, Zahra Mohamadnia^a*^, Ebrahim Ahmadib

^a^Department of Chemistry, Institute for Advanced Studies in Basic Science (IASBS), Gava Zang, Zanjan, 45137-66731, Iran

^b^Department of Chemistry, University of Zanjan, P.O. Box 45195-313, Zanjan, Iran

*Corresponding author e-mail address: [z.mohamadnia@iasbs.ac.ir](mailto:z.mohamadnia@iasbs.ac.ir), <https://orcid.org/0000-0002-1937-4980>, Tel: (+98) 24 3315-3130, Fax: (+98) 24 3315-3232

**1. Characterization of Cr-SNS-B and Cr-SNS-D complexes**

Bis(2-alkyl-sulfanyl-ethyl)-amine ligands, named SNS-B and SNS-D, were prepared and identified. SNS-B has a butyl group, while SNS-D has a dodecyl group. The preparation of these ligands is detailed in **Figure S1**.

**Fig. S1**. Provision of SNS-R tridentate ligands.

FT-IR, CHNS, ^13^C NMR, and ^1^H NMR analysis was conducted to identify and confirm the prepared ligands. Bis(2-decylsulfanyl-ethyl)-amine (SNS-D) was obtained as a pale oily liquid with an 80% yield. The chemical structure of this ligand is shown in **Figure S2**.

**Fig. S2.** Bis(2-dodecyl-sulfanyl-ethyl)-amine ligand (SNS-D).

The desired ligand was confirmed to have formed based on the ^1^H NMR nuclear magnetic resonance spectrum (**Figure S3**), which had a frequency of 250 MHz. In the deuterated chloroform (CDCl_3_) ^1^H NMR spectrum, there were seven peaks in the 0-3 ppm region. The triplet peak at 0.85 ppm indicated the presence of six hydrogen from the terminal methyl group (6H, t, CH_3_). The multiple peaks observed at 1.31-1.25 ppm were assigned to the hydrogen from the methylene groups of the carbon chain attached to the sulfur atom (SCH_2_CH_2_C_9_H_18_CH_3_). The hydrogen from the amine group (1H, -N-H) appeared as a broad peak at 1.9 ppm. The five peaks at 1.5 ppm corresponded to the two hydrogens from the group (4H, q, SC_2_H_4_CH_2_C_8_H_16_CH_3_). The methylene groups (4H, t, SCH_2_CH_2_C_10_H_20_CH_3_), (4H, t, NHCH_2_CH_2_S), and (4H, q, NHCH_2_CH_2_S) appeared as triplet peaks at 2.43 and 2.6 ppm, respectively. Additionally, a peak at 2.74 ppm was observed.


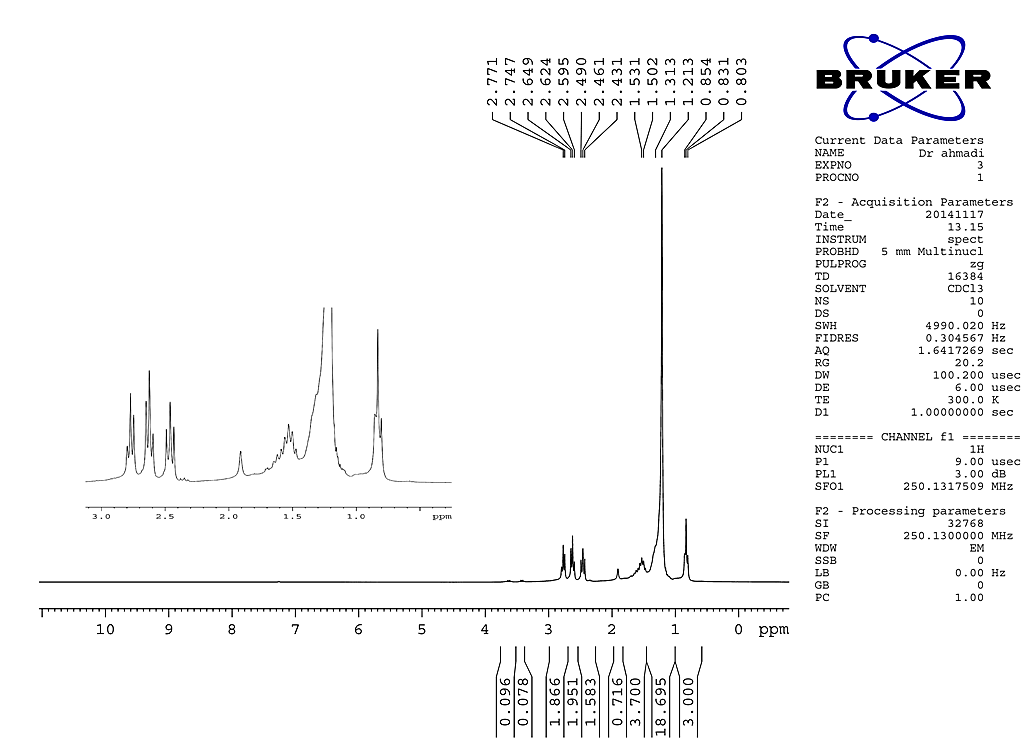


**Fig. S3.** ^1^H NMR spectrum of bis(2-dodecylsulfanyl-ethyl)-amine (SNS-D) ligand in CDCl_3_ solvent.

**Figure S4** shows the carbon nuclear magnetic resonance (^13^C NMR) spectrum of the SNS-D ligand at a frequency of 60 MHz. The peaks at 14.04 and 22.63 ppm correspond to the terminal methyl carbon (CH_3_) and the methylene carbon adjacent to it (CH_2_CH_3_). The carbons of the group SC_2_H_4_C_7_H_14_C_3_H_7_ exhibit peaks in the range of 29.28-48.74. The peaks at 32.86-01.32, 31.32, and 32.48 are associated with the (SC_2_H_4_C_7_H_14_CH_2_C_2_H_5_), (SCH_2_CH_2_NH), and (SCH_2_CH_2_NH) carbons, respectively.


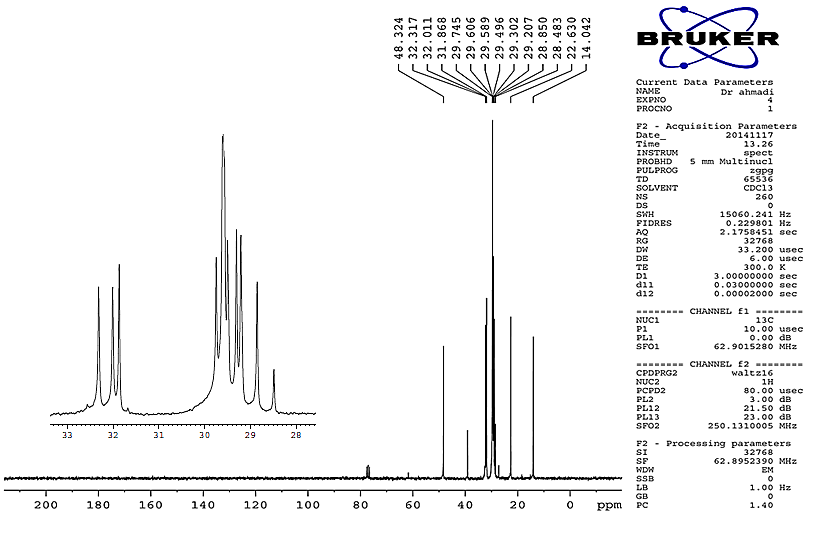


**Fig. S4.** ^13^C NMR spectrum of bis(2-dodecylsulfanyl-ethyl)-amine (SNS-D) ligand in CDCl_3_ solvent.

Also, the SNS-B ligand was a pale oily liquid with a 70% yield. The structure of this ligand is exposed in **Figure S5**.

**Fig. S5.** The structure of bis(2-butyl-sulfanyl-ethyl)-amine (SNS-B) ligand.

**Figure S6** shows the hydrogen nuclear magnetic resonance (^1^H NMR) spectrum of the SNS-B ligand with a frequency of 250 MHz. The hydrogen of terminal methyl group (6H, t, CH_3_) appear as a triple peak at 0.8 ppm. The methylene group attached to the terminal methyl (SCH_2_CH_2_CH_2_CH_3_) shows a five-fold peak at 1.38 ppm, while the methylene group (SCH_2_CH_2_CH_2_CH_3_) also shows a five-fold peak at 1.61 ppm. The peak at 1.9 ppm, with an integral equivalent to one hydrogen, corresponds to the amino group (N-H). The triple peaks at 2.30, 2.57, and 2.71 ppm, with an integral equivalent to 2 hydrogens, are related to the groups SCH_2_C_3_H_7_, SCH_2_CH_2_NH, and NHCH_2_.


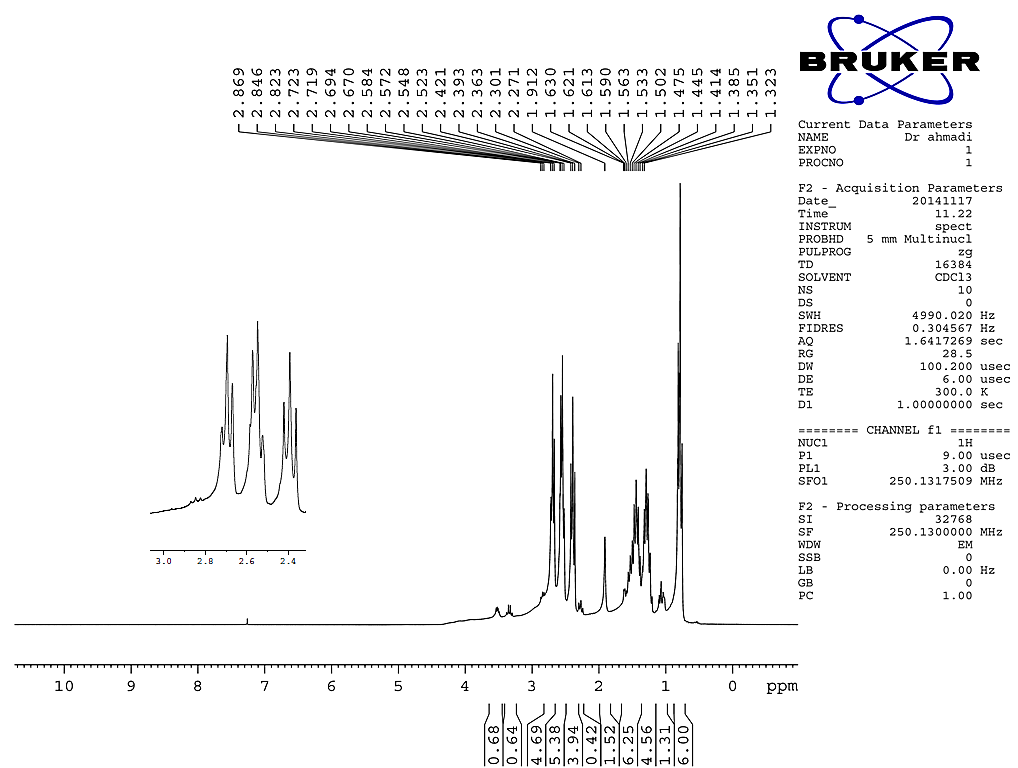


**Fig. S6.** ^1^H NMR spectrum of bis(2-butylsulfanyl-ethyl)-amine (SNS-B) ligand in CDCl_3_ solution.

The spectrum of the SNS-B ligand, taken at a frequency of 60 MHz, displays peaks at 13.56, 21.51, 31.56, 31.73, 21.32, and 48.24 ppm. These peaks correspond to CH_3_, CH_2_CH_3_, SCH_2_CH_2_C_2_H_5_, SCH_2_C_3_H_7_, SCH_2_CH_2_NH, and SCH_2_CH_2_NH carbons, as shown in **Figure S7**.


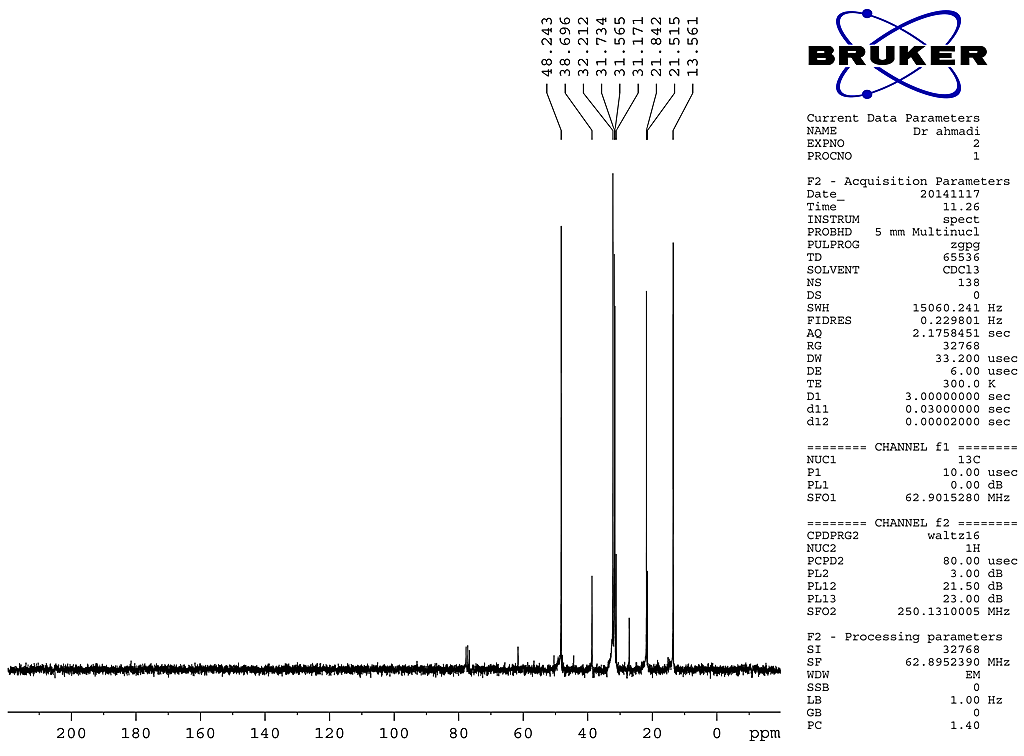


**Fig. S7.** ^13^C NMR spectrum of bis(2-butylsulfanyl-ethyl)-amine (SNS-B) ligand in CDCl_3_ solvent.

**Figure** **S8** shows the infrared spectra for the SNS-D and SNS-B ligands. The presence of the second type amine functional group (N-H) is indicated by the peak in the 3400-3300 cm^-1^ region. The peaks in the 2920-2960 cm^-1^ range correspond to the asymmetric stretching vibrations of C-H bonds, while the peaks at 2850 cm^-1^ are associated with the symmetric stretching vibrations of C-H bonds. The sorption band at 1465 cm^-1^ is related to C-N stretching vibrations. Additionally, the peaks at 1377 cm^-1^ are attributed to CH_3_ symmetric bending, and the sorption band in the region of 725 cm^-1^ indicates the presence of the C-S bond.


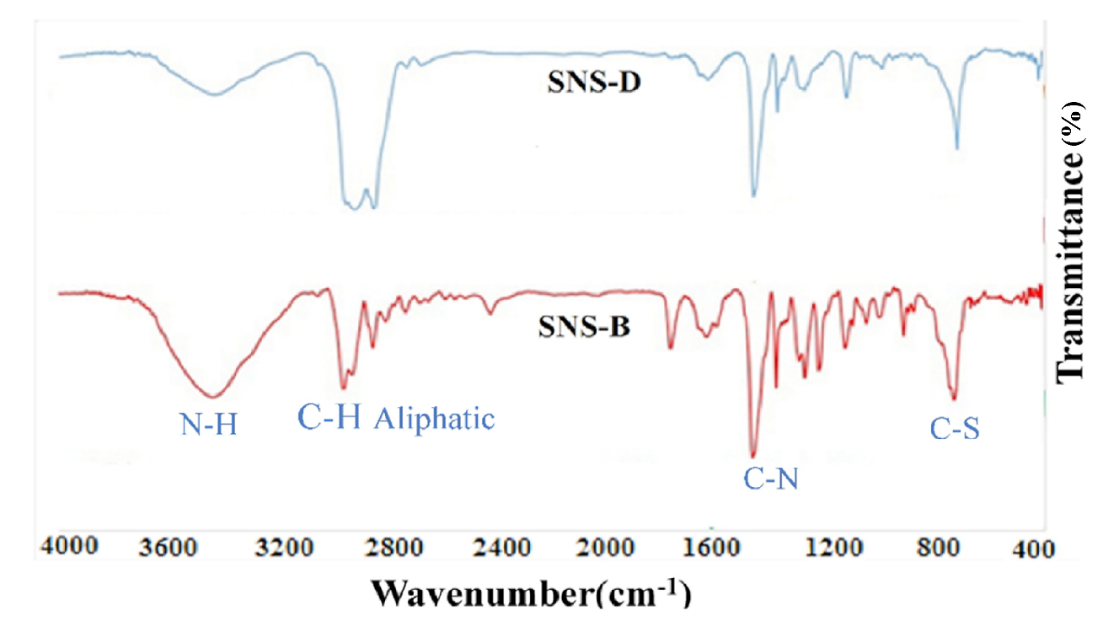


**Fig. S8.** FT-IR spectrum of SNS-D and SNS-B ligands in KBr tablets.

Furthermore, through elemental analysis, we have identified SNS-D and SNS-B ligands. The CHNS analysis data can be found in **Table S1**.

**Table S1.** Elemental analysis results of SNS-D and SNS-B ligands.

| Sample | %C | H% | N% | S% |
| --- | --- | --- | --- | --- |
| SNS-D (C_28_H_59_NS_2_) | (70.48)^b^  (70.97)^a^ | (13.21)  (12.58) | (3.30)  (2.95) | (13.01)  (13.50) |
| SNS-B (C_12_H_27_NS_2_) | (57.26)  (57.77) | (11.45)  (10.91) | (5.52)  (5.61) | (25.77)  (25.71) |

a: Theoretical data b: Experimental data

The ligands were synthesized and reacted with a chromium source, CrCl_3_.(THF)_3_, and a solvent called tetrahydrofuran. As a result, two catalysts, Cr-SNS-B and Cr-SNS-D, were obtained with yields of 60% and 80% respectively. The preparation method for the Cr-SNS-R complex is shown in **Figure S9**. When the ligand was introduced to the solution, it immediately turned green and formed a complex. This reaction took place at room temperature and proceeded quite rapidly, completing within 20 minutes.

**Fig. S9.** Preparing Cr-SNS-R complexes.

**Figure S10** shows the FT-IR spectrum of Cr-SNS-R complexes. According to the figure, a single peak at 3178 cm^-1^ indicates the presence of type II amine due to N-H stretching vibrations. The coordination of the ligand's N atom to chromium results in a shift in the absorption frequency of the N-H bond and the corresponding peak. The peaks in the range of 2942-2929 cm^-1^ confirm the presence of asymmetric stretching C-H bonds, while the peak at 2854 cm^-1^ confirms the presence of symmetric stretching C-H in the compound's structure. Additionally, the weak band at 1615 cm^-1^ corresponds to stretching Cr-Cl. The absorption band observed at 1460 cm^-1^ is related to C-N stretching vibrations. The bonds in the region of 1378 cm^-1^ are attributed to CH_3_ symmetric bending. The sorption band at 1600 cm^-1^ indicates the presence of the C-S bond. Lastly, the weak band at 1517 cm^-1^ corresponds to tensile Cr-N.


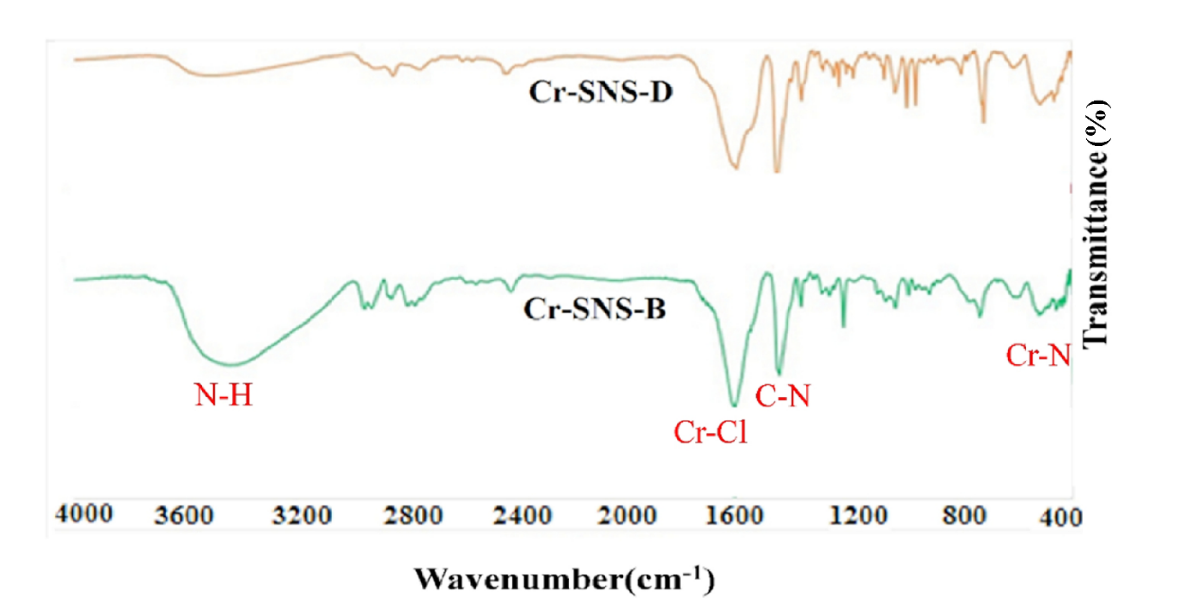


**Fig. S10.** FT-IR spectrum of Cr-SNS-R complexes in KBr tablets.

The complexes Cr-SNS-B and Cr-SNS-D were identified through elemental analysis. The information obtained from the CHNS analysis is provided in **Table S2**.

**Table S2.** Elemental analysis results of Cr-SNS-D and Cr -SNS-B complexes.

| Sample | %C | H% | N% | S% |
| --- | --- | --- | --- | --- |
| Cr-SNS-D (C_28_H_59_Cl_3_CrNS_2_) | (53.53)^b^  (53.19)^a^ | (9.63)  (9.41) | (1.97)  (2.22) | (10.56)  (10.14) |
| Cr-SNS-B (C_12_H_27_Cl_3_CrNS_2_) | (35.44)  (35.34) | (6.23)  (6.67) | (3.70)  (3.43) | (15.94)  (15.72) |

a: Theoretical data b: Experimental data

**2.3. Identification of ordered mesoporous silica (SBA-15)**

SBA-15 mesoporous silica was prepared using Stuckey's method. To determine the structural characteristics of SBA-15, various techniques including FT-IR, BET porosity method, X-ray diffraction (XRD), scanning electron microscope (SEM), and thermal gravimetric (TGA) were employed. **Figure S11** shows the FT-IR spectrum of the mesoporous silica. The observed peaks at 467 cm^-1^ and 800 cm^-1^ correspond to the symmetric and asymmetric Si-O-Si bond vibrations in the main chain of silica mesoporous. The sharp peak in the range of 1000-1200 cm^-1^ indicates the stretching vibration of the Si-O bond. Additionally, the broad peak in the range of 3000-13500 cm^-1^ is attributed to the presence of hydroxyl groups on the surface of SBA-15.

**Fig. S11.** FT-IR spectrum of SBA-15 species.

The nitrogen adsorption-desorption curve at 77 K shows a type IV isotherm with a relative pressure range of 0.6 to 0.8. This indicates a highly ordered mesoporous compound. The BET calculations yield a surface area of 1042 m^2^/g, an average volume of 1.20 cm^3^/g, and an average pore diameter of 4.63 nm. Additionally, the diameter of the holes determined through the nitrogen absorption branch, using the BJH method, is equal to 10.6 nm. **Figure S12** illustrates the sharpness of the BJH graph and the DH curve, further confirming the regular mesoporous nanostructure.

| \|   b) \|   a) \| \| --- \| --- \| \|   d) \|   c) \| |  |
| --- | --- | --- | --- | --- | --- |

**Fig. S12.** a) Nitrogen absorption-desorption curve of mesoporous silica substrate SBA-15, b) BET curve related to SBA-15, c) DH curve resulting from the nitrogen desorption branch of SBA-15, d) BJH curve resulting from the nitrogen absorption branch of SBA-15.

The X-ray pattern for SBA-15 mesoporous silica, with hexagonal symmetry P6mm, is depicted in **Figure S13**. This pattern exhibits three distinct diffraction peaks: (100), (110), and (200). The (100) peak, the most prominent one, has an interlayer distance of 87 angstroms, which corresponds to the parameter of the large unit cell (a_0_=10/3 nm).

**Fig. S13.** X-ray diffraction pattern for SBA-15 species.

**2.4 Characterization of Cr-SNS-R@SBA-15 catalyst**

**Figure S14** shows the FT-IR spectra of the Cr-SNS-B@SBA-15 and Cr-SNS-D@SBA-15 catalysts. The Cr-SNS complex exhibits peaks at 3280 cm^-1^, which correspond to N-H bonds. Additionally, peaks in the range of 2700-2900 cm^-1^ are associated with aliphatic C-H bonds, while peaks at 1400-1470 cm^-1^ indicate C-N symmetric stretching vibrations. The presence of peaks in the 600-700 cm^-1^ range indicates the presence of C-S bonds. Both catalysts show a peak in the 2850-2950 cm^-1^ region, which is indicative of aliphatic stretching C-H groups. These peaks confirm that the catalysts are stabilized on the substrate in a heterogeneous state.


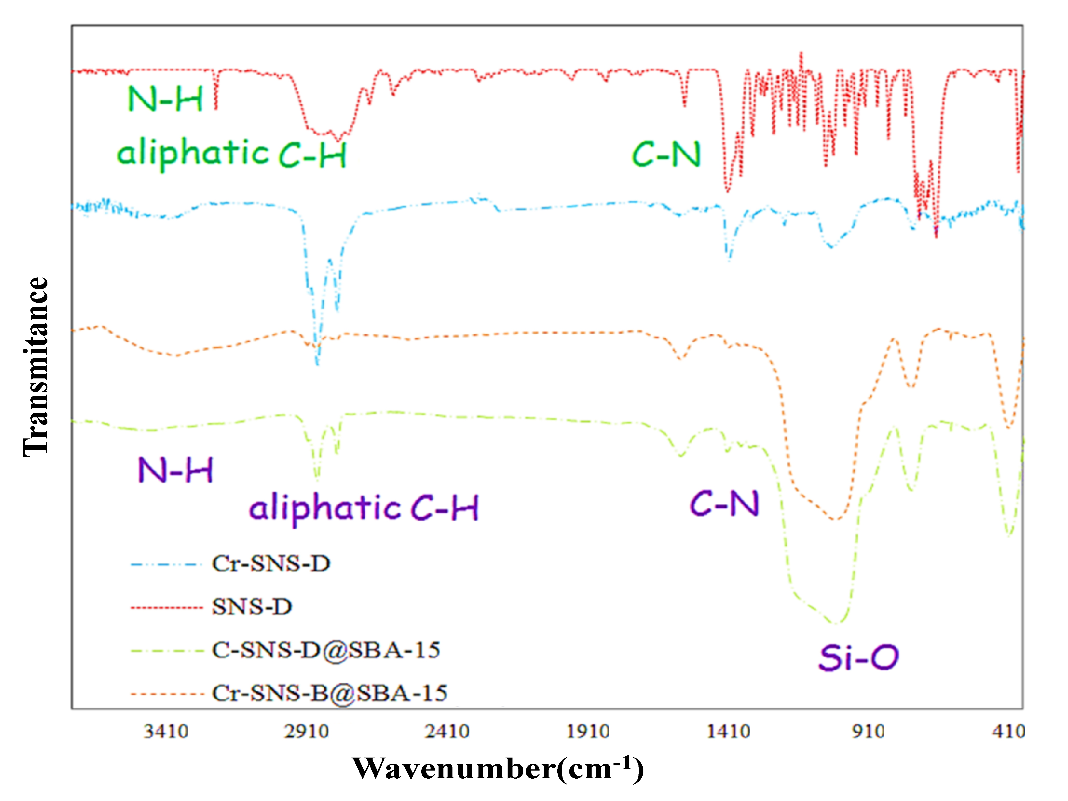


**Fig. S14.** FT-IR spectrum of heterogeneous catalysts Cr-SNS-B@SBA-15 and Cr-SNS-D@SBA-15.

The reflection-transmission UV-Vis spectroscopic analysis confirmed the stabilization of Cr-SNS-R complexes on the mesoporous silica substrate (**Figure S15**). The absorption peaks at 450 nm and 650 nm correspond to the d-d electron transitions of the metal. A similar pattern was observed comparing the spectrum of the catalysts prepared with the Cr-SNS-D complex. However, the peaks in the catalysts were slightly shifted to 437 nm and 640 nm. This pattern further confirmed that the oxidation number of chromium remained unchanged during the catalyst preparation process, and chromium remained as Cr (III) after stabilization.


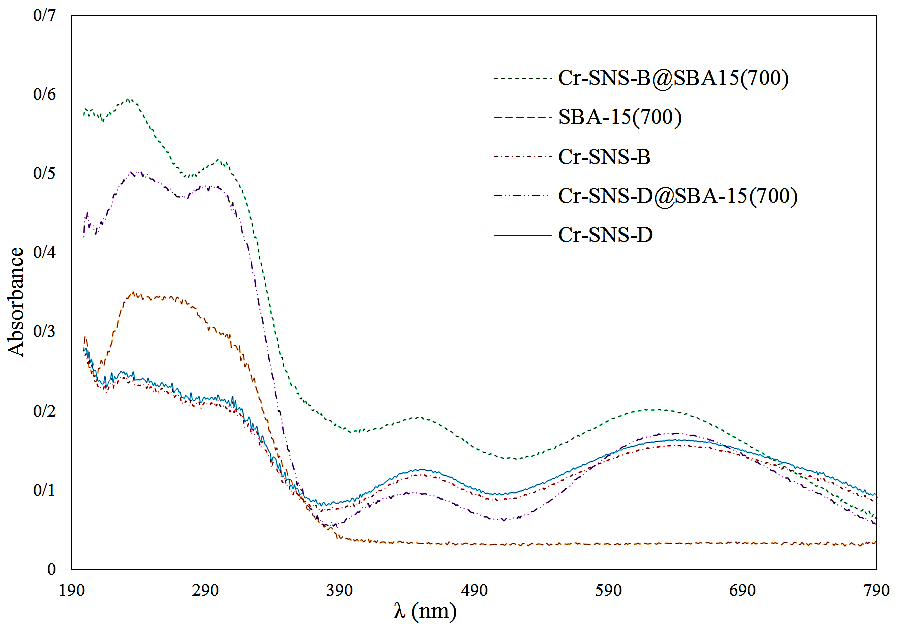


**Fig. S15.** UV-Vis spectroscopic analysis of reflection-transmission catalysts Cr-SNS-D@SBA-15 and Cr-SNS-B@SBA-15.

The catalytic thermal stability of Cr-SNS-D@SBA-15 was examined using thermogravimetric analysis (see **Figure S16**). The chart reveals an overall weight loss. The first weight loss (3.75) observed up to 120 °C is attributed to the removal of water and organic solvents. The subsequent reduction in the second, third, and fourth weights indicates an 8.43% decrease corresponding to the ligand attached to chromium. The final weight loss, which reaches 1.32% up to 800˚C, is associated with surface dehydroxylation. The loading of the Cr-SNS-D catalyst amounts to approximately 0.14 mmol/g of substrate.

**Fig. S16.** Thermal gravimetric analysis diagram of Cr-SNS-D@SBA-15 catalyst in a nitrogen atmosphere with a heating rate of 10°C/min.

The thermal stability of the Cr-SNS-B@SBA-15 catalyst was evaluated using thermal gravimetric analysis (**Figure S17**). The graph exhibits some overall weight loss. The first weight loss (approximately 5%) up to a temperature of 150 °C can be attributed to the removal of organic solvents used. The weight loss observed at 600-150 °C corresponds to 10.31% and is associated with the elimination of the organic components of the catalyst, specifically the SNS-B ligand. The final weight loss, occurring above 600 °C, is a consequence of surface dehydroxylation. The loading rate of Cr-SNS-B was determined to be approximately 0.29 mmol/g for the Cr-SNS-B@SBA-15 substrate. The catalyst loading with the SNS-B ligand is higher compared to SNS-D. This discrepancy can be attributed to the shorter length of the butyl chain in SNS-B, which allows for greater penetration into the silica cavities. Consequently, a greater decrease in surface area was observed in the porosimetry analysis.

**Fig. S17.** Thermal gravimetric analysis diagram of Cr-SNS-B@SBA-15 catalyst in a nitrogen atmosphere with a heating rate of 10 °C/min.
